# Associations between psychosis and visual acuity impairment: A systematic review and meta‐analysis

**DOI:** 10.1111/acps.13330

**Published:** 2021-06-15

**Authors:** Natalie Shoham, Michelle Eskinazi, Joseph F. Hayes, Gemma Lewis, Magnus Theodorsson, Claudia Cooper

**Affiliations:** ^1^ Division of Psychiatry University College London London UK; ^2^ Camden and Islington NHS Foundation Trust London UK; ^3^ Department of Ophthalmology King's College Hospital London UK

**Keywords:** Schizophrenia, psychotic disorders, visual acuity, myopia

## Abstract

**Objectives:**

Several theories propose that visual acuity impairment is associated with psychosis. Visual impairment could lead to psychosis or the converse, or they may share underlying pathology or risk factors. In the first evidence synthesis in this area for over 25 years, we collated studies measuring the association between visual acuity impairment and psychosis.

**Methods:**

We searched the MEDLINE, EMBASE, PsycINFO, and Web of Science databases for studies published from 1992 to 2020, using the Newcastle Ottawa Scale to assess risk of bias. We narratively synthesized findings and meta‐analyzed sufficiently homogenous results.

**Results:**

We included 40 papers, which reported on 31 studies. Evidence from seven cohort studies was inconsistent, which precluded meta‐analysis of this study design. These contradictory results also made it difficult to draw conclusions regarding a temporal association. We found evidence for an association from eight cross‐sectional studies treating visual acuity impairment as the exposure and psychosis as the outcome [pooled odds ratio (OR) =1.76, 95% confidence interval (CI): 1.34–2.31], and four with the reverse exposure and outcome (OR: 1.85, 95% CI: 1.17–2.92). Seven case–control studies with mixed findings were found, but only two primarily addressed our research question, and these findings were mixed.

**Conclusions:**

Although evidence supports a cross‐sectional association between visual acuity impairment and psychosis, further research is needed to clarify the temporal direction, given the mixed findings in cohort studies. Understanding the association may give insights into prevention strategies for people at risk of visual acuity impairment and psychosis.

**Summations**

- An association between psychosis and visual acuity impairment is well‐evidenced in cross‐sectional research, but findings from longitudinal studies give inconsistent results.
- Future research should focus on establishing the temporality of this association and elucidating the underlying mechanisms.

**Limitations**

- This review was limited to English language studies.
- The meta‐analyses from this review are based on observational studies, and results must therefore be interpreted with caution.
- We did not identify any studies investigating the longitudinal association between psychosis as exposure and visual impairment as outcome.

## 1 INTRODUCTION

There is a growing body of research exploring how visual acuity impairment and psychosis interrelate. Psychosis is a broad term encompassing illnesses such as schizophrenia and their associated symptoms, which include delusions, hallucinations, and thought disorder. In this review, we define visual acuity impairment as reduced measured acuity or the subjective experience of not seeing clearly.

An association between psychosis and impaired visual acuity has been demonstrated cross‐sectionally and longitudinally, including very large population‐based datasets.^1^, ^2^ It extends to both psychotic disorders and psychotic symptoms.^1^, ^3^ The relationship may be bidirectional. Psychotic disorders might lead to visual acuity impairment through reducing an individual's ability to seek help to maintain good vision, for example by attending optician appointments.^3^ Both psychotic illnesses and antipsychotic medications also increase risks of developing diabetes and cardiovascular disease, which can damage retinal vasculature and reduce eyesight.^4^, ^5^ Antipsychotic medications may directly cause blurred vision as an anticholinergic side effect,^6^ and symptoms such as disorganization could increase tolerance for blur.^7^ Nevertheless, studies showing that childhood ocular pathology is associated with increased risk of schizophrenia in adulthood imply that psychotic illnesses leading to reduced visual acuity cannot fully account for the association.^8^, ^9^

It is also speculated that aberrant visual input might be a potential cause of psychosis.^10^, ^11^ Visual hallucinations have been instigated through blindfolding healthy individuals.^12^ This phenomenon is well‐recognized in Charles–Bonnet syndrome, where loss of vision leads to visual hallucinations without other psychotic symptoms.^10^ The “Protection Against Schizophrenia” model posits that the effects of aberrant visual input on cortical NMDA receptors might cause psychosis, with absent and perfect vision being protective.^10^, ^11^ This model is based on an observed absence of any case reports of a congenitally cortically blind person developing schizophrenia, despite shared risk factors for these conditions.^13^ The mechanisms by which congenital or early blindness may be protective are not clear, but it is suggested that enhanced perceptual processing, greater working memory capacity, and prevention of abnormal visual input conferred by early blindness may play a role.^13^ Indeed, Silverstein et al. have described how many cognitive functions typically impaired in schizophrenia are in fact enhanced in congenitally blind individuals.^13^

A further possibility linking visual acuity impairment and psychosis is that common neuropathological processes cause both.^14^, ^15^ Retinal thinning has been associated with schizophrenia and neurodegenerative diseases such as Alzheimer's disease and may reflect a change in central nervous system function.^16^ Further, the offspring of people with psychotic illnesses, who have a higher risk of developing psychosis themselves, have been found to have altered retinal function.^17^, ^18^ Visual acuity impairment and psychotic illnesses could also share risk factors, for example, vitamin D deficiency.^19^, ^20^

The most recent literature review regarding sensory impairment and psychosis in older adults was conducted in 1993.^21^ It reported that evidence of association between visual acuity impairment and psychosis was inconsistent and highlighted methodological flaws in the existing research. Limitations included lack of appropriate control groups and unreliable measurement of visual acuity impairment.^21^

### 1.1 Aims of the study

We aimed to update and expand on this review, by conducting the first systematic review for over 25 years to explore the association between visual acuity impairment and psychosis across all age‐groups. We aimed to determine the strength of evidence for an association, and the direction of this relationship, to inform future research, and specifically work exploring the etiology of psychosis.

## 2 METHODS

This review was registered on PROSPERO (CRD42019129214).^22^

We used the OVID interface to search the databases MEDLINE, EMBASE, and PsycINFO on August 18, 2020, limiting studies to human subjects and English language. The databases Open Grey^23^ and Web of Science were also searched on August 19 and September 10, 2020, respectively. We combined search terms encompassing visual acuity impairment with terms related to psychosis. These are described in Supplement Material S1.

Inclusion criteria were as follows:

1. Quantitative studies of any design that compared psychotic symptoms or illnesses as the outcome in people with visual acuity impairment relative to people without, or
2. Quantitative studies of any design that compared visual acuity impairment as the outcome in people with psychotic symptoms or illnesses relative to people without.

We included research studies published from January 1, 1992, onwards where:

- Visual acuity was defined as either measured visual acuity or self‐reported visual clarity
- Psychosis was defined as either reporting psychotic symptoms, or diagnosis of psychotic disorder whether self‐reported or determined by psychiatric interview or from medical records.

Subjective impairment has previously been reported to be a suitable proxy for objective impairment.^24^

Exclusion criteria were as follows:

- Studies with fewer than 30 exposed participants, due to limited validity relating to low power to detect an association.^25^
- Studies reporting a measure of color blindness or visual processing but without a measure of visual acuity or self‐reported visual clarity.
- Studies that excluded participants with visual acuity worse than 20/20 on Snellen chart or equivalent.
- Studies that only measured visual hallucinations and no other psychotic symptoms. This criterion was added to avoid overestimating any effect due to studies focused on Charles Bonnet syndrome, which differs from psychotic disorders in that the hallucinations are an isolated symptom and full insight is retained.

We exported search results into EndNote^26^ and then Covidence^27^ to facilitate co‐screening and record keeping by separate reviewers. Duplicates were removed automatically using inbuilt duplicate detection software in these packages.

The first author (NS) screened the titles and abstracts of potential studies to determine inclusion, with a 10% random sample of records independently screened by ME. Eligibility of studies was subsequently confirmed by NS, and ME independently checked the full text of >20% of all retrieved articles. Disagreement was resolved through discussion and consensus between NS and ME, and, if necessary, CC or JH.

Risk of bias was assessed using the Newcastle Ottawa Scale (NOS) for cross‐sectional, case–control, or cohort studies by NS and ME independently.^28^, ^29^ Where assessing the association between visual acuity impairment and psychosis was not the primary aim of the study, the risk‐of‐bias score related to the quality of the study regarding measurement of this specific association. The authors agreed, regarding the interpretation of the NOS for cross‐sectional studies, that we did not require assessment of outcome to be blinded, if it was objective; studies to include a power calculation if the sample size was >1000, nor for studies to have established comparability between respondents and non‐respondents if the response rate was >90%. We defined a quality score of 7+ as indicating a low risk of bias, consistent with published systematic reviews.^30^, ^31^ We deemed studies with a score of <4 to have a high risk of bias. Disagreements regarding NOS score were resolved through discussion.

We prioritized studies with a low risk of bias in the narrative review. Where multiple relevant results were reported, we reported odds or hazard ratios with the most robust level of adjustment in the Forest plots.^32^, ^33^, ^34^ We report distance visual acuity impairment where both near and distance visual acuity impairments were reported on separately, for comparability with other studies and consistency with criteria for certifiable visual impairment.^35^ Similarly, we chose schizophrenia when multiple psychotic disorder diagnoses were assessed, for comparability. We reported findings according to study type. Where there were enough studies rated as at low risk of bias, we compared studies of older and younger adults to account for the burden of psychosis in older adults potentially having different etiology, such as neurodegenerative disease.^36^ For the cross‐sectional studies, we also compared studies that reported only on psychotic symptoms and those that included psychotic diagnoses, and studies that used objective or subjective measures of visual impairment.

We summarized the level of evidence using the Evidence Based Medicine Consult guidelines, where.

- A = consistent evidence from randomized‐controlled trials
- B = consistent evidence from observational studies
- C = extrapolations from observational studies at higher risk of bias
- D = troublingly inconsistent evidence from studies at any level.^37^, ^38^

We specified in advance that we would conduct meta‐analysis if three or more studies with low risk of bias could be combined.^22^ We used random‐effects meta‐analysis to account for differences between study designs. This type of analysis includes a measure of estimated between‐study heterogeneity in the weighting, to avoid giving an overly precise estimate in the presence of heterogeneity.^39^ If a compatible effect estimate was not reported but could be calculated from raw data, we did this. We combined cross‐sectional studies that reported an odds ratio. We treated studies that used visual acuity impairment as exposure and psychosis as outcome and the converse separately, since these odds ratios are not theoretically interchangeable when adjusted. We used fully adjusted odds ratios where possible, due to evidence and guidelines, suggesting that this is likely to obtain the least‐biased pooled estimate.^32^, ^33^, ^34^ We also separately combined unadjusted odds ratios where these were provided or could be calculated, as a sensitivity analysis to reduce heterogeneity. We reported the *I* ^2^ statistic to describe the proportion of variation in results caused by heterogeneity. We classed 25% as low heterogeneity, 60% as moderate, and 75% as high.^40^ Data were analyzed in STATA version 16.^41^

## 3 RESULTS

### 3.1 Search results

**FIGURE 1:**
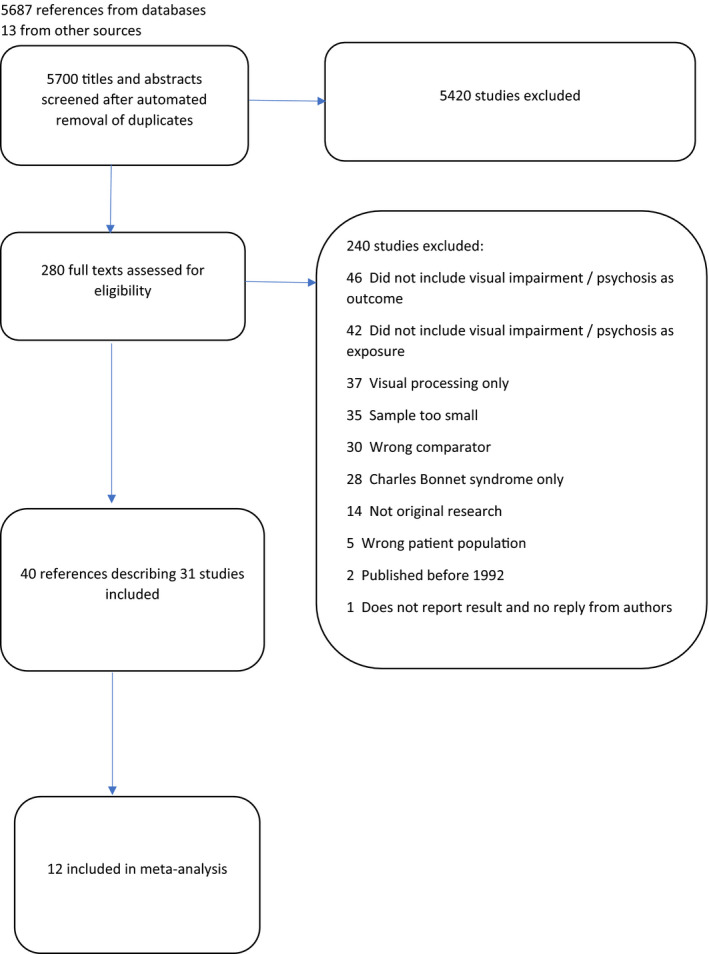
PRISMA diagram. 5687 references from databases. 13 from other sources. [Colour figure can be viewed at wileyonlinelibrary.com]

NS screened all 5700 titles and abstracts for inclusion, and ME co‐screened 570 (10%) (Figure 1). Cohen's kappa coefficient for inter‐rater reliability was >0.8, with agreement for >99% abstracts. 280 full texts were screened, of which ME co‐screened 65 (23%), giving Cohen's kappa of 0.63, with 88% agreement. The reasons for exclusion of full texts are shown in the PRISMA diagram (Figure 1). ME also checked data extraction from four (10%) of studies, with complete agreement. Forty papers that reported on 31 studies were finally included in the review. Emailing four experts in the field did not identify any additional studies.

### 3.2 Description of studies

Included studies comprised a total of 7,369,169 participants ranging from 16 to 102 years of age. We identified seven cohort studies, seven case–control studies, and 11 cross‐sectional studies, which reported the relationship between psychosis as an outcome and visual acuity impairment as exposure. Relatively few studies reported on the converse relationship, and all were cross‐sectional in design (*n* = 6).

Eleven studies restricted analysis to older age‐groups, by using cutoffs of age 50 or older, or by recruiting from facilities primarily for older adults.^42^, ^43^, ^44^, ^45^, ^46^, ^47^, ^48^, ^49^, ^50^, ^51^, ^52^ Two cohort studies investigated visual problems in childhood.^8^, ^9^

In total, 19 (61%) studies had a low risk of bias,^1^, ^2^, ^3^, ^8^, ^9^, ^46^, ^47^, ^49^, ^50^, ^51^, ^52^, ^53^, ^54^, ^55^, ^56^, ^57^, ^58^, ^59^, ^60^ while an additional two met low risk‐of‐bias criteria for cross‐sectional but not longitudinal results.^44^, ^61^ All other studies were rated as having a moderate risk of bias.^21^, ^42^, ^43^, ^45^, ^62^, ^63^, ^64^, ^65^, ^66^, ^67^

### 3.3 Cohort studies

**FIGURE 2:**
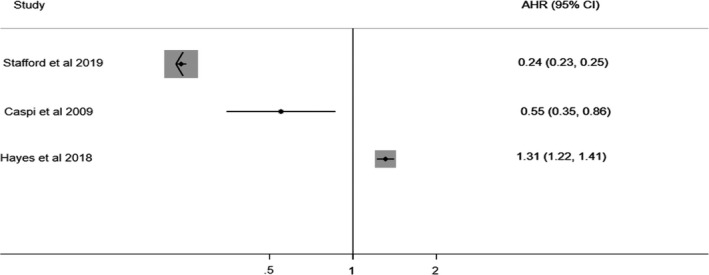
Cohort Studies Reporting on risk of Psychotic Illness in Visual Impairment. AHR, adjusted hazard ratio; 95% CI, 95% confidence interval.

Seven cohort studies were identified^2^, ^8^, ^9^, ^44^, ^48^, ^53^, ^54^ (Table 1, Figure 2). Collectively, they reported on over 4,830,050 participants. All reported the relationship between psychosis as an outcome and visual acuity impairment as exposure.

**TABLE 1:** Cohort studies reporting on psychosis in visual impairment.

Study and country	Population	Sample size (exposed participants)	Exposure	Outcome	Maximum length of follow‐up (years)	Factors adjusted for in results shown	Results (for people with exposure relative to people without)	Risk‐of‐bias rating
Hayes et al. 2018 Sweden^2^	Male military conscripts aged 18–19 from 1974 to 1997	1,140,710 (84,663 mild visual impairment 62,678 moderate visual impairment 90,142 severe visual impairment)	Snellen chart acuity, both corrected uncorrected, recorded as decimal where 20/20 vision =1.0	Inpatient diagnosis of schizophrenia or other non‐affective psychotic disorder from linked hospital records	38 (Mean 24.75)	Age, year of interview, SES, IQ History of CMD, parental SMI, alcohol use disorder, substance use disorder	For uncorrected acuity <1.0 AHR schizophrenia: 1.31, 95% CI 1.22–1.41 AHR other psychotic illness: 1.17, 95% CI 1.08–1.26 For best corrected acuity <1.0 AHR any psychotic illness: 1.21, 95% CI 1.15–1.2	Low
Caspi et al. 2009 Israel^53^	Unselected population of Israeli‐born male adolescents aged 16–17	678,674 (40,201)	Refractive error based on best corrected visual acuity measured using Snellen chart	Inpatient diagnosis of schizophrenia from linked hospital records	NR	Intelligence, years of education, SES	AHR 0.55, 95% CI 0.35–0.8	Low
Stafford et al. 2019 Sweden^54^	Whole population sample of adults aged 60+ from national registers for psychiatric illness, followed from 1980	Total =3,007,378 (2037)	Visual impairment according to National Patient Register	Very late‐onset schizophrenia‐like psychosis, defined as ICD diagnosis of non‐affective psychotic disorder since 1980 recorded in National Patient Register.	31	Age, sex, age–sex interaction, offspring with non‐affective psychosis, region of origin, birth period, disposable income, death of child, death of partner, hearing impairment	AHR 0.24, 95% CI 0.23–0.25	Low
Schubert et al. 2005 Sweden^9^	“High‐risk” sample of offspring of women with psychosis and matched controls born in 1973–1977.	Total =110 52 high risk offspring 58 controls	Severity of visual dysfunction aged 4 measured by visual acuity at age 4 or referral to specialist due to vision problems before age 4	Diagnosis of schizophrenia spectrum disorder made using SCID	18	–	OR 16.07 95% CI 1.85–139.60, *p* = 0.003	Low
Schiffman et al. 2006 Denmark^8^	All children born in one hospital from 1959 to 1961 whose parent had a specialist diagnosis of schizophrenia. Controls whose parent had another psychiatric diagnosis Controls with no parental psychiatric diagnoses	Total =242 at follow‐up From initial cohort of 265: 90 offspring of a parent with schizophrenia 93 offspring of a parent with another psychiatric diagnosis 82 offspring of parents with no psychiatric diagnoses	Composite eye examination score which included eye alignment and related deficits, suppression, depth perception, pursuit movements, and visual acuity (measured using the STYCAR Vision Test at ages 11–13 and categorized as normal or abnormal). Higher scores indicate worse visual function.	Diagnosis of schizophrenia spectrum disorder made by psychiatrist when participants were aged 31–33 using SCID and PSE; or hospital records.	20	‐	Schizophrenia spectrum group mean eye score =147.90 Comparison group mean eye score =118.32 *p* = 0.035	Low
Hamedani et al. 2020 USA^48^	Data from two longitudinal studies were analyzed: NHATS: a nationally representative study of medicare beneficiaries aged 65 or older.	NR	Distance and near vision, and blindness assessed using yes/no questions.	Proxy‐reported visual or auditory hallucinations	7	Age, sex, ethnicity, income, hypertension, diabetes, smoking, stroke, education, depression, anxiety, dementia, hearing loss	AOR in near‐vision impairment 1.77 95% CI 1.43–2.17 AOR in distance vision impairment: 1.74 (1.43–2.11) AOR in blindness: 1.62 (0.98–2.68)	Medium
	HRS: a nationally representative survey of US adults over the age of 50	NR	Overall eyesight, distance vision, and near vision assessed using questions.	Proxy‐reported visual or auditory hallucinations	12	Age, ethnicity, sex, income, hypertension, diabetes, smoking, stroke, education, nursing home status, physical functional impairment, hearing loss	AOR in overall visual impairment: 1.40 95% CI 1.22 – 1.60 AOR in near‐vision impairment: 1.42 (1.23–1.63) AOR in distance vision impairment: 1.63 (1.41–1.87) AOR in blindness: 1.79 (1.10–2.92)	Medium
Blazer et al. 1996 USA^44^	Community sample of older adults over 65, identified using four‐stage stratified sampling design from census	2936	Visual deficit measured on a continuous scale based on six questions.	Paranoia measured using CES‐D	3	NR	AOR 1.32 95% CI 1.02–1.71 *p* < 0.05	Medium

SES, socioeconomic status; IQ, intelligence quotient; CMD, common mental disorder; SMI, serious mental illness; AHR, adjusted hazard ratio; 95% CI, 95% confidence interval; NR, not recorded; ICD, International Classification of Diseases; SCID, Structured Clinical Interview for DSM‐III‐R; OR, odds ratio; PSE, Present State Examination; AOR, adjusted odds ratio; NHATS, The National Health and Aging Trends Study; HRS, The Health and Retirement Study; CES‐D, Center for Epidemiological Studies Depression Scale.

#### 3.3.1 Studies classed as at low risk of bias

The 5/7 cohort studies rated as having a low risk of bias^2^, ^8^, ^9^, ^53^, ^54^ recruited three distinct populations: young male military conscripts,^2^, ^53^ older people,^54^ and children.^8^, ^9^

Two very large studies explored whether, in young male military conscripts, refractive error predicted future diagnosis of psychotic illness.^2^, ^53^ Both measured visual acuity with Snellen charts and used linked hospital records to determine subsequent psychotic illness diagnosis status, but they found opposing results. A Swedish study of >1 million young men^2^ found that worse visual acuity increased the risk of psychotic illness, while an Israeli study^53^ of >650,000 young men found that it reduced the risk of schizophrenia. We noted some key differences between these studies. The Israeli study focused exclusively on schizophrenia and assessed corrected visual acuity.^53^ It did not state length of follow‐up or describe the measure of visual acuity impairment in detail, and reported a lower prevalence of myopia than another study using the same data.^68^ The Swedish study assessed multiple measurements of the exposure including uncorrected visual acuity and additionally tested non‐affective psychotic disorder as an outcome.^2^ It included a sensitivity analysis excluding participants who developed psychosis within five years of the exposure measurement to ensure prevalent psychosis was not driving the findings, which were robust to this.^2^

A study of older adults used Swedish national registry data from >3 million people aged 60 in 1980 and investigated whether visual acuity impairment predicted diagnosis of very late‐onset schizophrenia‐like psychosis (VLOSLP) up to 31 years later.^54^ Contrary to the authors’ hypothesis, visual acuity impairment predicted a significantly lesser likelihood of being diagnosed with VLOSLP. The authors comment that this finding was unexpected and suggest that using register‐based diagnoses may have led to artificial evidence of negative association as people with psychotic illness can be less able to access health care and are therefore less likely to have visual acuity impairment recorded.^54^ They may also be more likely to be subject to “diagnostic overshadowing”, where physical complaints are wrongly attributed to psychiatric illness.^54^ Further, survivor bias is possible, as participants who received a diagnosis of psychosis earlier in life were not included.^54^

Two smaller studies (*n* = 242 and *n* = 110) examined children including offspring of parents with psychotic illness and matched comparators.^8^, ^9^ Both found that ophthalmic problems in childhood were associated with a future diagnosis of schizophrenia spectrum disorder. These studies measured visual acuity at ages 4^9^ and 11–13^8^ and followed children up for 18 and 20 years, respectively.

There were only two cohort studies in similar populations that reported results compatible with combination in meta‐analysis,^2^, ^53^ and as these results were contradictory, it was not appropriate to combine them.

#### 3.3.2 Moderate and higher risk‐of‐bias studies

The 2/7 cohort studies classed as having moderate risk of bias reported data on older adults and found that visual acuity impairment was associated with subsequent psychotic symptoms.^44^, ^48^

### 3.4 Case–control studies

There were seven case–control studies, reporting data on 723 people aged over 18^21^, ^55^, ^63^, ^64^, ^65^, ^66^, ^69^ (Table 2). These all compared people with a diagnosed psychotic illness to controls without psychosis. Two investigated differences in visual acuity impairment as a primary aim,^21^, ^62^ with the remainder assessing acuity purely to establish comparability between groups in visual processing or retinal thickness experiments.^55^, ^63^, ^64^, ^65^, ^66^, ^70^

**TABLE 2:** Case–control studies reporting odds of visual impairment in psychosis.

Study and country	Cases	Controls	Sample size Total (Cases)	Outcome	Results	Risk‐of‐bias rating (for outcome of interest)
Lee et al. 2013 Malaysia^55^	Consecutive patients with schizophrenia attending a secondary care center. Diagnosis based on psychiatric examination and DSM‐IV‐TR criteria. People with myopia >2.0 diopters excluded.	Hospital staff and volunteers matched for age, sex, and ethnicity. Psychiatric disorders were excluded using SCID	60 (30)	Best corrected visual acuity measured with Snellen chart and refraction	Patient mean visual acuity score: 100.00 Control mean visual acuity score: 102.17 No statistically significant difference between groups using independent t test (*p* = 0.068)	Low
Prager and Jeste 1993 USA^21^	Patients with schizophrenia aged 45+ recruited primarily from Veterans Affairs Clinic. Diagnosis confirmed using SCID. Organic mental disorder was excluded by investigation.	Comparison group with no major psychopathology recruited from other studies at the clinic.	Total =87 16 with late‐onset schizophrenia 25 with early‐onset schizophrenia 20 with mood disorder	Near‐vision acuity measured using Lebensohn chart. Distance‐vision acuity measured using Snellen chart, both with and without correction. Groups compared using Kruksal–Wallis test	Uncorrected near‐visual acuity: no group differences. Corrected near‐visual acuity: All psychiatric groups had worse acuity than controls. The differences reached significance for left eye and binocular vision. Uncorrected distance visual acuity: no group differences Corrected distance visual acuity: all psychiatric groups had worse mean acuity. The differences reached significance for patients with early‐onset schizophrenia on left eye and binocular vision and for mood disorder patients on left eye vision. Significance level *p* < 0.05	Medium
Cumurcu et al. 2015 Turkey^62^, ^69^	Patients with DSM‐IV‐TR schizophrenia diagnosis aged 18–65 evaluated at the Eye Outpatient Clinic. All had been treated with an antipsychotic medication for 2+ years and had no medical comorbidity.	Patients visiting the same institution matched for age, sex, and education	130 (70)	Visual acuity measured by Snellen chart	No evidence of difference in incidence of refractive error between the two groups using two‐sided t test (*p* = 0.082).	Medium
Brittain et al. 2010 UK^63^, ^70^	Patients with a DSM‐IV diagnosis of schizophrenia recruited from outpatient and long term assisted living settings Diagnosis was confirmed by their treating clinician, chart review, and SCID. People with best corrected visual acuity <0.8 decimal were excluded.	Control status was determined using the psychotic screening SCID. Potential control subjects were excluded if any of their first‐degree relatives had a history of psychotic illness.	129 (64)	Best corrected visual acuity measured using Freiburg visual acuity test.	Patient mean visual acuity: 1.31. Control mean visual acuity: 1.33 This difference was not statistically significant at *p* < 0.05 level using t test	Medium
Keane et al. 2019 USA^64^, ^72^, ^73^	People aged 18–65 with first‐episode psychosis or schizophrenia/schizoaffective disorder assessed using SCID or electronic medical record. People with visual acuity poorer than 20/32 were excluded.	Controls without 4‐year college degrees were preferentially recruited. Controls had no diagnosis of any psychotic or mood disorder, no current psychotropic medication, and no first‐degree relative with schizophrenia or schizoaffective disorder.	120 49 with schizophrenia or schizoaffective disorder 23 with first‐episode psychosis	Visual acuity measured using LogMAR chart In‐house visual acuity correction kit used when necessary	Using ANOVA, the group with schizophrenia/schizoaffective disorder had poorer visual acuity than controls (*p* < 0.001) and people with a first episode of psychosis (*p* < 0.05)	Medium
Schechter et al. 2005 USA^65^	Patients with schizophrenia or schizoaffective disorder recruited from a state psychiatric facility. Diagnosis was confirmed by chart review, consultation with physicians and SCID. Participants with visual acuity <20/32 were excluded	Healthy volunteers with no history of SCID defined psychiatric disorder, neurological or ophthalmologic disorders, alcohol or substance dependence within the last six months or abuse within the last month.	106 (57)	Visual acuity measured using ETDR chart	Patients had poorer mean visual acuity assessed using t test: Patient mean 0.88 Control mean 1.07 *p* < 0.001	Medium
Silverstein et al. 2014 Denmark^66^	Patients aged 18–60 and diagnosed with schizophrenia or first‐episode psychosis, referred to the study by mental health inpatient staff. All patients were receiving antipsychotic medication. Diagnoses were confirmed by SCID. Groups were matched on visual acuity, but between‐group acuity was still tested due to small differences.	Healthy controls without diagnosable lifetime psychiatric conditions (confirmed using SCID); no use of psychotropic medication over the preceding 6 months, and no first‐degree relatives with psychotic illness.	91 22 with first‐episode psychosis 34 with schizophrenia	Measured visual acuity in LogMAR units.	The groups did not differ in acuity using ANOVA.	Medium

SCID, Structured Clinical Interview for Diagnostic and Statistical Manual (DSM); DSM, Diagnostic and Statistical Manual; IQ, intelligence quotient; LogMAR, logarithm of minimal angle resolution; ETDR, early treatment diabetic retinopathy; ANOVA, analysis of variance.

All case–control studies measured objective visual acuity impairment using Snellen, LogMAR, Freiburg, or similar charts. The Freiburg test is a computerized visual acuity test.^71^ Five studies allowed some degree of correction of impairment using aids during the measurement,^21^, ^55^, ^63^, ^64^, ^66^, ^72^, ^73^ and two did not state whether correction was employed.^65^, ^69^ The number of participants in each study ranged from 60 to 130.

Of note, four studies excluded participants with myopia above a certain level,^55^, ^63^, ^64^, ^65^ one broadly matched groups by visual acuity (but still tested for a difference),^66^ and one selected controls from an eye clinic.^62^ This likely limited the ability of case–control studies to detect an association.

#### 3.4.1 Studies classed as at low risk of bias

Only one study was rated as having low risk of bias.^55^ This study reported on a group of 30 adults with schizophrenia attending a secondary care center and 30 matched controls.^55^ It excluded people with myopia requiring lenses greater than 2.0 diopters (classed as mild myopia^74^). There was a lower mean visual acuity score in the schizophrenia group compared with the control group, but this was not strongly supported by statistical testing (*p* = 0.068).

#### 3.4.2 Studies classed as at moderate or higher risk of bias

Three of the six case–control studies at moderate risk of bias found evidence of lower visual acuity in the groups with psychotic illness for at least some types of vision,^21^, ^65^, ^72^ while the remaining three found no difference between the groups.^62^, ^63^, ^66^ One study found that the lower visual acuity applied to people with established schizophrenia, but not people with first‐episode psychosis.^64^

### 3.5 Cross‐sectional studies

**FIGURE 3:**
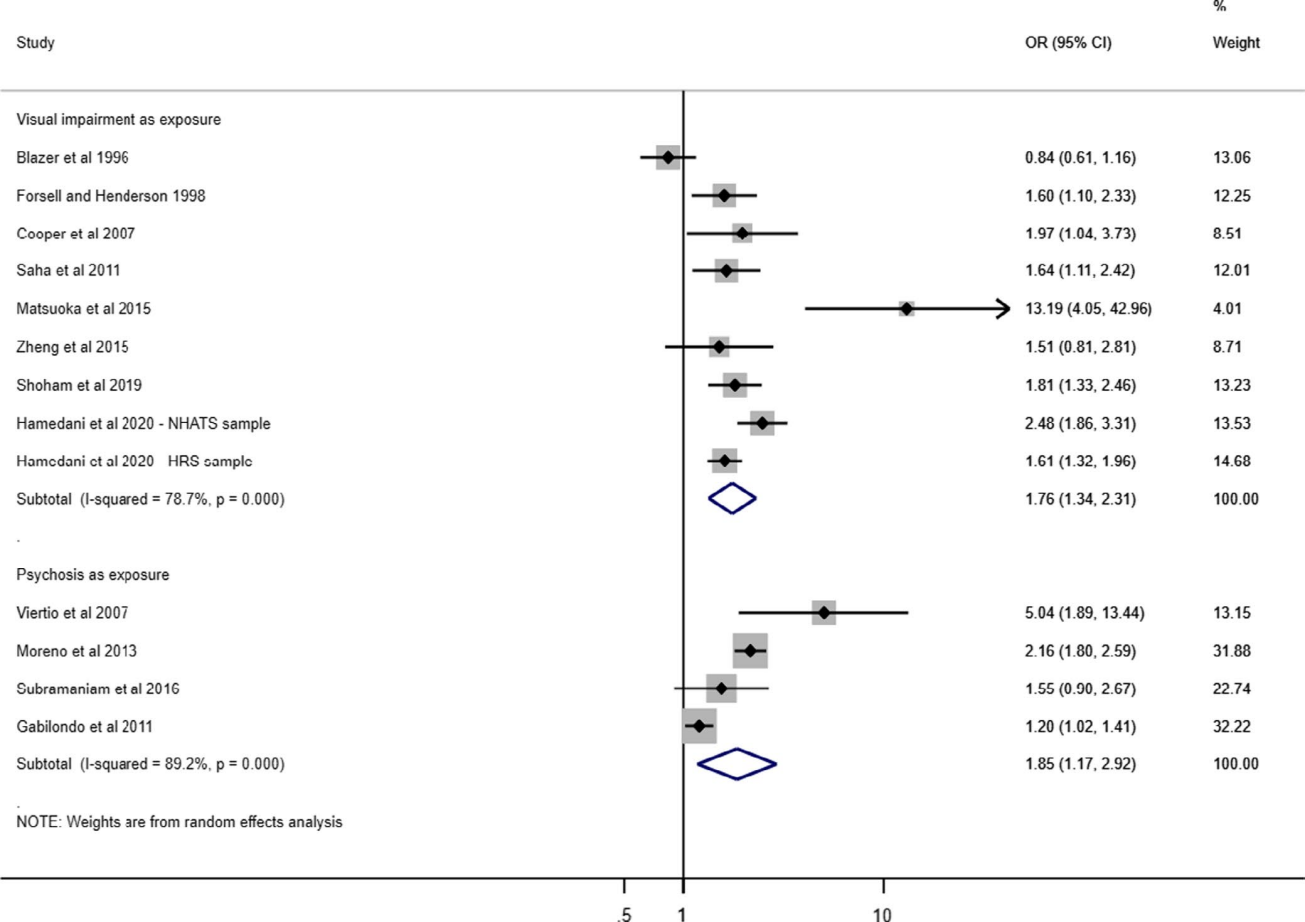
Cross‐Sectional Studies Reporting Association between Psychosis and Visual Impairment. OR, Odds Ratio (with most robust adjustment); 95% CI, 95% Confidence Intervals; NHATS, The National Health and Aging Trends Study; HRS, The Health and Retirement Study. [Colour figure can be viewed at wileyonlinelibrary.com]

Nineteen studies reported on the cross‐sectional association between visual acuity impairment and psychosis^1^, ^3^, ^42^, ^43^, ^44^, ^45^, ^46^, ^47^, ^48^, ^49^, ^50^, ^51^, ^52^, ^56^, ^57^, ^58^, ^59^, ^60^, ^67^ (Tables 3 and 4, Figure 3). These covered a total of 2,541,332 people aged over 16. Two studies reported data both cross‐sectionally and longitudinally: Only the cross‐sectional data are included here.^44^, ^48^

**TABLE 3:** Cross‐sectional studies reporting on association between psychosis and visual impairment in people of any age.

Study and country	Sample	Year of data collection	Sample size (number of exposed participants)	Exposure	Outcome	Factors adjusted for	Results (for exposed relative to unexposed participants)	Risk‐of‐bias rating
Saha et al. 2011 Australia^56^	General population household survey of people aged 16 to 85	2007	8771 (593)	Positive response to the question: have you ever had sight problems lasting more than 6 months?	Possible psychosis based on CIDI	Age, sex, marital status, migrant status, alcohol/drug abuse, anxiety disorder, depressive disorder, family history of psychosis	AOR 1.64, 95% CI 1.11–2.41, *p* < 0.01	Low
Shoham et al. England^57^	Nationally representative household sample of people aged 16+	2014	7107 (934)	Self‐reported difficulty reading a newspaper or seeing a face across the room, even with visual aids.	Psychotic symptoms elicited by PSQ.	Age, sex, ethnicity, employment, education, housing, AUDIT score	AOR 1.81, 95% CI 1.33–2.44, *p* < 0.001.	Low
Zheng et al. 2015 China^58^	Patients aged 18+ consecutively admitted to a psychiatric center 44% schizophrenia diagnosis; 33% bipolar affective disorder diagnosis; 23% major depressive disorder.	2013	Total =356 (87)	Presenting visual acuity measured by LogMAR chart with spectacles, if required. Distance visual impairment defined as LogMAR score ≥0.5	Severity of psychotic symptoms on BPRS Raw numbers with visual impairment provided for each diagnostic category	–	For schizophrenia relative to other diagnoses: OR 1.51 95% CI 0.81–2.82**˩** No association between mean BPRS score and distance visual impairment *p* = 0.63	Low
Cooper et al. 2007 Scotland^60^	Community sample: all persons aged 16+ known to their GP with intellectual disability within a defined region	NR	1020	The C21st Health Check includes Kay's pictures and caregiver report	Diagnosis of psychosis made by a psychiatrist in people who scored positive on PAS‐ADD	Age, gender, level of ability previously having lived in a long‐stay hospital, special communication needs, epilepsy, smoking, type of accommodation/support.	AOR 1.97 95% CI 1.04–3.74 *p* = 0.038	Low
Viertio et al. 2007 Finland^3^, ^83^, ^84^	Nationally representative population survey of people aged 30+	2000 – 2001	Total sample =6588 (56 schizophrenia, 72 other non‐affective psychosis, 38 affective psychosis)	SCID diagnosis in people reporting diagnosis of psychotic disorder/possible psychotic or manic symptoms on CIDI, at interview, or from hospital case notes.	Visual Acuity on LogMAR chart and near‐vision chart with usual visual aids. Distance visual impairment defined as acuity <20/40	Age, sex	Schizophrenia: Distance vision impairment: AOR 5.04 95% CI 1.89–13.48 *p* < 0.001 Near‐vision impairment: AOR 6.22 95% CI 2.61–14.82 *p* < 0.001 Other non‐affective psychosis or affective psychosis: no evidence of association	Low
Moreno et al. 2013 Stubbs et al. 2016 Koyanagi et al. 2016 Multinational^1^, ^85^, ^86^	World Health Survey Data: randomly selected household sample of people aged 18+ across 70 countries Moreno et al: sample from 52 countries. Stubbs et al: sample from 48 low‐ and middle‐income countries. Koyanagi et al: sample from 44 low‐ and middle‐income countries. Excluded people with lifetime diagnoses of psychotic disorders or who reported psychotic experiences in the absence of depression.	2002 – 2004	Moreno et al 224,254 (NR) In Stubbs et al 242,952 (NR) Koyanagi et al 195,479 (2.7% subsyndromal depression, 3.0% brief depressive episode, 7.1% depressive episode).	Psychotic symptoms elicited from CIDI 3.0 Self‐reported diagnosis of psychotic illness	Self‐reported presence (yes/no) of vision problems	Sample weighting was applied. Koyanagi et al: Age, sex, wealth, education, alcohol consumption, anxiety, country	**Moreno et al:** In people with psychotic symptoms but no diagnosis: OR 1.67, 95% CI 1.59–1.75 In people with psychotic symptoms and psychosis diagnosis: OR 2.16, 95% CI 1.80 to 2.58 Stubbs et al: Evidence of association between both visual impairment and psychosis diagnosis and visual impairment and subclinical psychosis (*p* < 0.0001). Koyanagi et al. Linear regression coefficient for vision problems in people with depression only relative to people with depression and psychotic experiences was −0.05, 95% CI: 1.72 to 2.61	Moreno and Stubbs: Low Koyanagi et al: Medium
Gabilondo et al. 2017 Spain^59^	Everyone registered in Population Stratification Programme (healthcare dataset covering population of Basque country)	2011	2,255,406 (7731)	Healthcare records: diagnosis of schizophrenia (F20, ICD10) made by a mental health specialist in a public mental health resource.	Diagnosis of blindness or low vision in healthcare records	Age, sex, deprivation Index	AOR 1.20 95% CI 1.02–1.42, *p* = 0.032	Low
Kinoshita et al. 2009 USA^67^	Household survey of people aged 18+	2001 to 2003	2322 (85)	Visual impairment elicited by asking: [Do you have] a vision problem that prevents you from reading a newspaper even when wearing glasses or contacts?	Auditory hallucinations elicited using CIDI	Sex Stratification by age	AOR 2.16, 95% CI 0.87–5.33, *p* = 0.10 The association was significant in people aged 18–39: AOR 13.25, 95% CI 2.99 to 58.75, *p* < 0.001.	Medium

CIDI, Composite International Diagnostic Interview; AOR, adjusted odds ratio; 95% CI, 95% confidence intervals; PSQ, Psychosis Screening Questionnaire; AUDIT, Alcohol Use Disorders Test; BPRS, Brief Psychopathological Rating Scale; OR, odds ratio; **˩**, calculated from raw numbers by authors; SCID, Structured Clinical Interview for DSM‐IV‐TR; CIDI, Composite International Diagnostic Interview; LogMAR, logarithm of minimal angle of resolution; NR, not recorded; C21st, 21st century; PAS‐ADD, Psychiatric Assessment Schedule for Adults with Developmental Disabilities Checklist; NR, not recorded.

**TABLE 4:** Cross‐sectional studies reporting on association between visual impairment and psychosis in older adults.

Study and country	Sample	Year of data collection	Sample size (number of exposed participants)	Exposure	Outcome	Factors adjusted for	Results (for exposed relative to unexposed participants)	Risk‐of‐bias rating
Livingston et al. 2001 England^49^	Household sample of people aged 65+	NR	720 (137)	Uncorrected visual impairment elicited by asking: “Do you have any problems with your sight?”; and whether this had been adequately corrected.	Perceptual distortion and affective response to delusions or hallucinations elicited from GMSE	Analysis repeated restricted to people with dementia	OR 2.8 *p* < 0.02 When analysis was restricted to people with dementia: OR 3.9 *p* < 0.05	Low
Subramaniam et al. 2016 Singapore^51^, ^87^	Population‐based study of people aged 60+	2011	2166 2.7% with paranoid ideation 2.8% with persecutory ideation 2.7% with hallucinations	Presence of paranoid ideation, delusions, and hallucinations assessed by GMSE	Eyesight problems elicited from WHO Disability Assessment Schedule	Sociodemographic variables (specifics not given)	Paranoid ideation: AOR 1.4 95% CI 0.6–2.9 *p* = 0.432 Persecutory delusions: AOR 1.3 95% CI 0.6–2.7 *p* = 0.550 People with hallucinations: AOR 2.1 95% CI 1.01–4.2 *p* = 0.046 Any of these symptoms: AOR 1.55 95% CI 0.9–2.7 *p* = 0.121	Low
Ballard and Bannister 1995 England^52^	People aged 65+ with mild or moderate dementia and informant contact at least weekly, recruited from consecutive referrals to old‐age psychiatry services	NR	124 (83)	Psychotic symptoms elicited using Burns’ Symptom Checklist	Visual defects according to CAMDEX, or participants who were registered blind or partially sighted or having been informed by a doctor that they could be.		Visual impairment was significantly associated with psychotic symptoms using Wald's test, *p* = 0.02	Low
Matsuoka et al. 2015 Japan [Letter]^50^	Consecutive outpatients aged 60+ seen at department of psychiatry between April 2009 and March 2013.	2009–2013	979 (157)	Visual impairment defined as poor visual capacity in the clinical examination and daily life, based on reports from participants and their caregivers	ICD10 diagnosis of psychosis occurring after age 60	Age, gender, hearing impairment	OR 13.19; 95% CI 4.05–43.00, *p* < 0.001	Low
Forsell and Henderson 1998 Sweden^46^	Community sample of people aged 75+ All residents in the region, including people living in institutions.		1220	Visual problems assessed by physicians as causing clinical distress	Paranoid ideation elicited through PRS	Cognitive dysfunction	OR 1.6 95% CI 1.1–2.0. This was “not significant” after controlling for cognitive dysfunction.	Low
Hamedani et al. 2020 USA^48^	NHATS: a nationally representative sample of people aged 65+	2002 to 2014	1520	Distance vision impairment defined as self‐reported difficulty seeing someone across the street.	Proxy‐reported hallucinations ascertained by asking “Does he or she ever see or hear things that are not there?”	Age, sex, ethnicity, income, hypertension, diabetes, smoking, stroke, education, depression, anxiety, dementia, hearing loss	In near‐vision impairment: AOR 1.77, 95% CI 1.32–2.39. In distance vision impairment: AOR 2.48, 95% CI: 1.86 to 3.31 In blindness: AOR 2.05, 95% CI: 0.88–4.78)	Low
	HRS: nationally representative survey of people aged 50+		3682	Overall eyesight, distance vision, and near vision assessed using scales	Proxy‐reported hallucinations ascertained by asking “Does he or she ever see or hear things that are not there?”	Age, sex, ethnicity, income, hypertension, diabetes, smoking, stroke, education, nursing home status, physical functional impairment, hearing loss	In impaired overall eyesight: AOR 1.32 95% CI 1.08–1.60 In distance vision impairment: AOR 1.61 95% CI 1.32–1.96 In near‐vision impairment: AOR 1.52 95% CI 1.25–1.85 In blindness: AOR 1.99, 95% CI: 0.94–4.19	Low
Blazer et al. 1996 USA^44^	Community sample of adults aged 65+ identified using four‐stage stratified sampling design from census	NR	3869	Visual deficit measured on continuous scale based on 6 questions	Paranoid symptoms elicited using CES‐D	Age, sex, ethnicity, marital status, education, income, ADLs, functional limitations, mobility, social network, social interaction, negative life events, depressive symptoms, cognitive impairment	AOR 0.84, 95% CI 0.61–1.14, *p* > 0.05	Low
Henderson et al. 1998 Australia^47^	Sample drawn from the electoral roll for Canberra, aged 70+	1990 to 1991	935	Psychotic symptoms elicited using questions from CIE.	Scale for visual impairment based on respondent report and interviewer observations.		Mean visual impairment score 7.8 in group with psychosis Mean visual impairment group 7.2 in group without psychosis. *p* = 0.07	Low
Bayón and Sampedro 2017 Spain^43^	Patients examined consecutively in cognitive neurology clinic. 607 had mild cognitive impairment or dementia. Subjects aged 65+ accounted for 80.5% of the total.	NR	Total =843 13.3% had recorded visual changes	Clinical records noting visual changes interfering with functional capacity in patients whose vision could not be corrected with lenses.	Delusions or hallucinations recorded in clinical notes.		Delusions and hallucinations were more prevalent in people with visual changes (*p* < 0.01)	Medium
Bazant et al. 2003 [Conference abstract] USA^42^	People presenting for geriatric assessment. Over half had dementia. Mean age 79	1997–2000	447	Visual acuity assessed using LNVAT. Impairment defined as score <20/40.	Hallucinations and delusions assessed using NPI Clinical diagnosis of psychosis		Multivariate analysis showed visual Impairment <20/60 level to be associated with hallucinations (OR =3.17) and impairment <20/40 to be associated with delusions (OR =1.85). Visual acuity at all levels failed to meet significance threshold with respect to clinical diagnosis of psychosis.	Medium
Ostling and Skoog 2002 Sweden^45^	Residents of Gothenburg aged 85 selected from census by systematic sampling	1986–1987	305 (58)	Delusions, hallucinations, or paranoid ideation elicited using CPRS, triangulated with informant interview and medical records.	Visual deficits that interfered with conversation and execution of tasks as observed at psychiatric examination.		Hallucinations: OR 3.4; 95% CI 1.0–11.1 Paranoid ideation: OR 3.6; 95% CI, 1.2–10.5 Delusions: OR 1.4 95% CI 0.2–6.9	Medium

GMSE, Geriatric Mental State Examination; OR, odds ratio; 95% CI, 95% confidence Intervals; WHO, World Health Organization; AOR, adjusted odds ratio; CAMDEX, Cambridge Mental Disorders of the Elderly Examination; ICD, International Classification of Diseases; NR, not recorded; CPRS, Comprehensive Psychopathological Rating Scale; NHATS, The National Health and Aging Trends Study; HRS, The Health and Retirement Study; CES‐D, Center for Epidemiological Studies Depression Scale; CIE, Canberra Interview for the Elderly; LNVAT, Lighthouse Near Visual Acuity Test; NPI, Neuropsychiatric Inventory; CPRS, Comprehensive Psychopathological Rating Scale.

#### 3.5.1 Studies classed as at low risk of bias (*n* = 15)

Fifteen out of nineteen studies were classified as having low risk of bias.^1^, ^3^, ^44^, ^46^, ^47^, ^48^, ^49^, ^50^, ^51^, ^52^, ^56^, ^57^, ^58^, ^59^, ^60^

Five of these investigated general population samples including adults of any age^1^, ^3^, ^56^, ^57^, ^59^; seven investigated community samples of older adults^44^, ^46^, ^47^, ^48^, ^49^, ^51^, ^52^; one a community sample of adults with intellectual disability^60^; and two recruited patients from psychiatric facilities, of which one focused on older adults.^50^, ^58^ Two had very large samples (>200,000 participants).^1^, ^59^

In determining the presence of visual acuity impairment, three studies used formal measures including LogMAR chart and Kay's pictures^3^, ^58^, ^60^; two used undescribed standardized physical examinations^51^, ^52^; one used diagnosis of blindness or low vision in healthcare records^59^; three used judgments from clinicians or carers^46^, ^47^, ^50^; and six used self‐report.^1^, ^44^, ^48^, ^49^, ^56^, ^57^

Ten studies investigated psychotic symptoms rather than diagnoses.^44^, ^46^, ^47^, ^48^, ^49^, ^51^, ^52^, ^56^, ^57^, ^58^ Two used either psychotic symptoms or diagnosis^1^, ^3^; three used diagnoses (from clinical records or research interview).^50^, ^59^, ^60^

In total, 11/15 studies found evidence of a positive association between visual acuity impairment and psychosis,^1^, ^3^, ^46^, ^48^, ^49^, ^50^, ^52^, ^56^, ^57^, ^59^, ^60^ with adjusted odds ratios (AORs) ranging from 1.20 to 13.19 (Figure 2). This included 5/8 studies of older adults^48^, ^49^, ^50^, ^51^, ^52^ and 6/7 studies of adults of any age.^1^, ^3^, ^56^, ^57^, ^59^, ^60^

### 3.6 Studies including adults of any age

#### 3.6.1 Objective measures of visual impairment

Four of the seven studies including younger adults used objective measures of visual acuity impairment, either LogMAR test score, Kay's pictures, or a diagnosis of blindness or low vision in clinical records.^3^, ^58^, ^59^, ^60^ They include the only study of younger adults, which did not find evidence of association at the *p* < 0.05 level.^58^ This took place in a psychiatric facility and was relatively smaller than other studies (*n* = 356). The point estimate for the odds ratio for visual acuity impairment in schizophrenia relative to other diagnoses was still suggestive of an association.^58^

#### 3.6.2 Subjective measures of visual impairment

Three studies including younger adults reported the association between self‐reported sight difficulty and psychosis.^1^, ^56^, ^57^ All found evidence of association, including one international study with over 2 million participants.^1^ Adjusted odds ratio point estimates ranged from 1.64 to 2.16.

### 3.7 Studies that recruited older adults

#### 3.7.1 Objective measures of visual impairment

Only 1/8 studies of older adults used an objective measure of visual acuity impairment.^52^ This measured (either) visual defects according to examination, or participants having been informed by a doctor that they could be registered as blind or partially sighted.^52^ The study found an association between visual acuity impairment and psychotic symptoms.

#### 3.7.2 Subjective measures of visual impairment

Seven older adult studies used self‐report or carer report of reduced vision.^44^, ^46^, ^47^, ^48^, ^49^, ^50^, ^51^ Four reported a significant, positive association between visual acuity impairment and psychotic symptoms ^46^, ^48^, ^49^, ^50^; one found evidence of association with hallucinations but not other psychotic symptoms^51^; one found a small difference in visual acuity impairment scores between groups with and without psychosis at the 0.05 < *p* < 0.1 level^47^; and one found no evidence of association.^44^

#### 3.7.3 Psychotic diagnosis vs psychotic symptoms

Of all 15 cross‐sectional studies with low risk of bias, 7/9 that reported exclusively on psychotic symptoms found evidence for an association with visual acuity impairment at the level *p* < 0.05^46^, ^48^, ^49^, ^51^, ^52^, ^56^, ^57^ compared with 5/6 that reported on psychotic illness diagnoses.^1^, ^3^, ^50^, ^59^, ^60^

### 3.8 Studies at moderate or higher risk of bias

Three out of four studies at moderate risk of bias found evidence of association between visual acuity impairment and psychosis: two in older adults^42^, ^43^ and one in adults.^67^ The other study found an association between visual acuity impairment and paranoid ideation, but not delusions or hallucinations.^45^

### 3.9 Meta‐analysis

We combined results for the twelve cross‐sectional studies with low risk of bias that reported an odds ratio or allowed one to be calculated, dividing these according to whether they treated visual acuity impairment (*n* = 8) or psychosis (*n* = 4) as the exposure (Figure 3).^1^, ^3^, ^44^, ^46^, ^48^, ^50^, ^51^, ^56^, ^57^, ^58^, ^59^, ^60^ We included two separate samples reported in one study.^48^ We used fully adjusted odds ratios where possible.^32^, ^33^ The meta‐analysis gave a pooled odds ratio where visual acuity impairment was the exposure of 1.76 (95% CI: 1.34–2.31), and for psychosis as exposure of 1.85 (95% CI: 1.17–2.92). Heterogeneity was high in both groups: *I* ^2^ statistic = 78.7%, *p* < 0.001, and 89.2%, *p* < 0.001, respectively. In view of this, we tested heterogeneity in subgroups of studies with adults and older adults. We found that where visual acuity impairment was the exposure, there was no evidence of heterogeneity among younger adult studies (*I* ^2^ = 0, *p* = 0.920), but heterogeneity remained high in older adult studies (*I* ^2^ = 89.1%, *p* < 0.001). Even after excluding two outlying older adult studies,^44^, ^50^ heterogeneity remained moderate. The pooled OR for younger adult studies was 1.74 (95% CI: 1.40–2.15). For older adult studies, it was 1.87 (95% CI: 1.18–2.98) (Figure 3). There were too few studies to test subgroups when the exposure was psychosis.

There was no strong evidence of publication bias from a Funnel plot or Egger's test (*p* = 0.386 where visual acuity impairment was the exposure, and *p* = 0.593 where psychosis was the exposure) (Figure S1).

As a sensitivity analysis, we also combined unadjusted odds ratios from six studies that provided sufficient information^1^, ^46^, ^48^, ^57^, ^58^, ^59^, ^60^ (Figure S2). This gave a pooled OR of 2.07 (95% CI: 1.38–3.11) and did not reduce heterogeneity (*I* ^2^ = 95.2%, *p* < 0.001).

### 3.10 Summary of evidence

We found grade D (troublingly inconsistent) evidence for an association between objectively measured visual acuity impairment as an exposure and schizophrenia as an outcome in longitudinal studies. We found no longitudinal studies that investigated the converse relationship (psychosis as an exposure and visual acuity impairment as an outcome).

We also found grade D (troublingly inconsistent) evidence for an association between visual acuity impairment and psychosis from case–control studies.

We found grade B (consistent evidence from observational studies) evidence, however, for a cross‐sectional association between visual acuity impairment, whether measured objectively or subjectively, and psychosis. This applied to both younger and older adults, and to psychotic diagnosis and symptoms.

## 4 DISCUSSION

Most studies (22/31) reported an association between impaired visual acuity and greater risk or symptoms of psychosis in at least one analysis.^1^, ^2^, ^3^, ^8^, ^9^, ^21^, ^42^, ^43^, ^44^, ^45^, ^46^, ^48^, ^49^, ^50^, ^51^, ^52^, ^56^, ^57^, ^59^, ^64^, ^65^ This was also true of most (15/21) that were rated as at low risk of bias.^1^, ^2^, ^3^, ^8^, ^9^, ^46^, ^48^, ^49^, ^50^, ^51^, ^52^, ^56^, ^57^, ^59^, ^60^ The evidence for a cross‐sectional association was strong and consistent and applied across the lifespan, and in studies where psychotic symptoms and visual impairment were professionally diagnosed or self‐reported.

There is some suggestion from two studies that the association may be larger for schizophrenia than for other psychotic illness diagnosis.^2^, ^64^ Arguably, this could support the hypothesis that visual impairment is a consequence rather than a risk factor for psychosis, because schizophrenia is typically associated with poorer functioning than other diagnoses and therefore more likely to impair eye care.^75^ Alternatively, it could support a hypothesis that visual impairment is specifically a risk factor for schizophrenia.^13^

The positive associations between visual impairment and symptoms were greater for hallucinations than delusions in three studies, which separated these out,^42^, ^45^, ^51^ raising the possibility that visual hallucinations partially drove the associations seen. We were, however, unable to separate these from other types of hallucination.

For a longitudinal relationship between visual acuity impairment as exposure and psychosis as outcome, evidence was conflicting in adult populations.^2^ Both (small, cohort) studies of children found evidence of association^8^, ^9^; suggesting that ophthalmic problems during a critical developmental phase may be implicated on the causal pathway to a future diagnosis of psychosis.

The discrepancy in findings between cross‐sectional and longitudinal studies warrants further attention. It could suggest that psychosis leads to visual impairment, rather than the converse. No studies have yet tested the longitudinal association between psychosis as exposure and visual acuity impairment as outcome. It might also be that psychosis and visual impairment co‐occur and are not causally associated. Perhaps visual acuity impairment reflects brain aging,^76^ and its impairment reflects faulty neural processing as much as faulty refraction. Nevertheless, given that the most robustly conducted longitudinal study found a temporal association, which was strongest for corrected acuity,^2^ the hypothesis that visual acuity impairment could be an etiological factor contributing to the development of psychosis remains plausible. The pooled cross‐sectional odds ratios are larger than those for some established risk factors for schizophrenia, for example, obstetric complications and birth seasonality^77^, ^78^; but smaller than or similar to those for others, such as having an affected parent or childhood trauma.^78^, ^79^

Regardless of whether a causal relationship exists between visual impairment and psychosis, the evidence to date suggests that clinicians caring for people with psychotic illnesses should be alert to the increased chance that their patients will have impaired visual acuity. Facilitation of optical testing could improve eye care for this group.^3^ Wider uptake might also mean that complications of comorbidities associated with psychotic illnesses such as diabetes are detected earlier, preventing sight loss.^4^, ^5^ Similarly, clinicians caring for people with visual impairment should be aware of the potential for mental illness, so that patients can be signposted to appropriate support when needed.

### 4.1 Strengths and limitations

Our systematic review is the first to bring together studies of visual acuity impairment and psychosis across the lifespan. We searched multiple databases and incorporated a wide variety of designs. We included studies where the visual acuity impairment–psychosis relationship was not the primary focus of the study.

Limitations exist in the included studies. Clearly, randomized‐controlled trials are not possible in this area, so all studies were observational. The strength of our findings is inevitably dependent on the methodology of the included studies. While we found several large, cohort studies at low risk of bias, their findings were conflicting. This may be due to measurement differences, differences in severity of visual acuity impairment, or different lengths of follow‐ups. Limitations make it difficult to draw conclusions from the case–control studies. Consequently, we concluded that the evidence from study designs that might allow interpretation of the direction of association between visual acuity impairment and psychosis was inconsistent and that it is not possible to surmise from this review whether a potential causal relationship exists.

There was statistical evidence of high heterogeneity between studies, except for four studies investigating a cross‐sectional association between visual acuity impairment as exposure and psychosis as outcome in adults of any age.^56^, ^57^, ^58^, ^60^ The high heterogeneity is to some extent expected given the variation in study settings, and exposure and outcome measures.

A key limitation of the cross‐sectional studies was that most used subjective measures of visual acuity such as asking participants whether they had eyesight difficulties, and the majority also relied on self‐reported psychotic symptoms. No cross‐sectional studies report adjusting for antipsychotic medication use, which may affect associations, in primary analyses.

A limitation of the two studies of children is that visual acuity was combined with other measures to make an overall visual examination score, and not reported on independently, so we cannot draw conclusions as to whether it was impaired visual acuity that drove the association with subsequent psychotic illness.

The findings of Hayes et al. in the Swedish study suggest that visual acuity impairment that cannot be corrected is the greatest risk factor; the association may therefore have been obscured by studies that did not employ optimal correction.^2^

There were also limitations of the review methodology. It is possible that studies reporting no association may have been missed due to not being indexed in databases, although the funnel plot and Egger's test did not show evidence of publication bias. We did not collate studies that used objective measures of visual processing, such as visual evoked potentials or electroretinograms, although this has been done previously.^16^ We were unable to search for studies that were not written in or translated into English. Some search terms, for example, ‘blind’, were not included due to having alternative meanings (such as allocation concealment) that would bring up a very large number of irrelevant studies. Nevertheless, hand‐searching reference lists and contacting experts aimed to ensure that any relevant studies not captured in the original search were still detected. We were unable to co‐screen all abstracts and studies, but the high concordance between reviewers in the subset co‐screened suggests that doing so would have had minimal impact on the results of the review. We attempted to contact authors for additional details regarding some studies and frequently did not receive replies. There is some evidence that inter‐rater reliability on the Newcastle–Ottawa Scale (NOS) is lower than ideal, but we aimed to mitigate this by discussing the NOS for all studies.^80^ We decided not to include studies that excluded participants with vision above the 20/20 level, but in doing so could have missed a small number of studies that measured group differences within the “normal” range.^81^ Finally, meta‐analysis of observational data should be interpreted with caution, given the tendency for variation in study designs and unmeasured confounding.^82^

## 5 CONCLUSIONS

Overall, current evidence supports the existence of a cross‐sectional association between visual acuity impairment and psychosis across age‐groups. Since the largest and highest quality cohort studies have found contradictory evidence regarding the longitudinal association between visual acuity impairment and subsequent psychotic illness; however, the issue is far from resolved. The possibility of psychosis leading to subsequent visual acuity impairment has not been explored longitudinally. Future research should focus on exploring potential bidirectional longitudinal associations using objective measures of visual acuity impairment and psychosis, and in different degrees of visual acuity impairment.

## DECLARATION OF INTEREST

Natalie Shoham plans to continue to research the association between visual impairment and psychosis for her NIHR‐funded PhD.

### PEER REVIEW

The peer review history for this article is available at https://publons.com/publon/10.1111/acps.13330.

## Supporting information

Figure S1

Figure S2

Table S1

Supplementary Material

## Data Availability

No new data generated.
