# Supplementary figures and images for: Associations between psychosis and visual acuity impairment: A systematic review and meta‐analysis
Source: Acta Psychiatr Scand. 2021 Jun 15;144(1):6–27. doi: 10.1111/acps.13330 (PMC8504204; doi:10.1111/acps.13330)

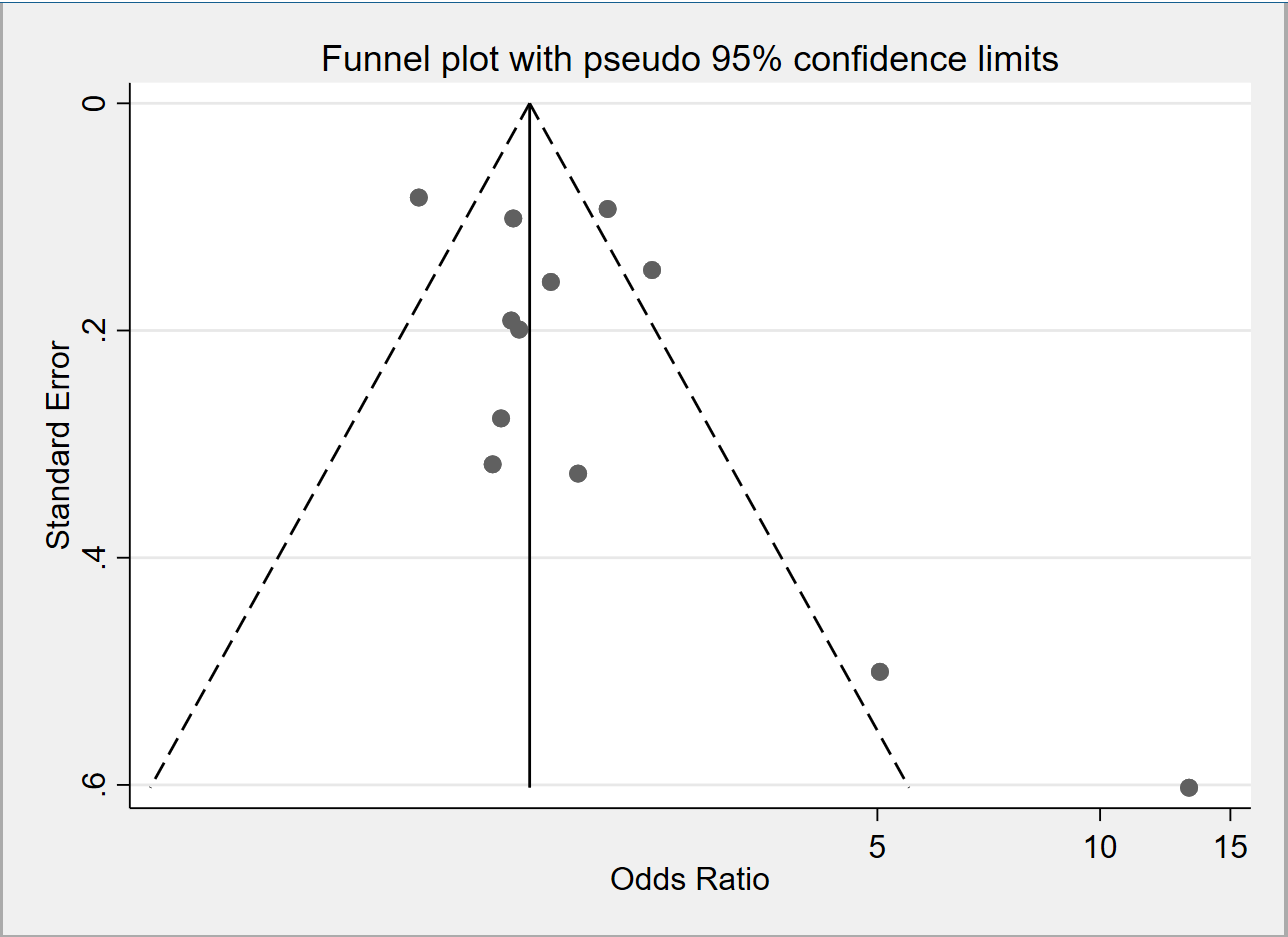

Supplement: Supplementary file 1 — Figure S1 [file ACPS-144-6-s003.png]
