# Supplementary material for: Associations between psychosis and visual acuity impairment: A systematic review and meta‐analysis
Source: Acta Psychiatr Scand. 2021 Jun 15;144(1):6–27. doi: 10.1111/acps.13330 (PMC8504204; doi:10.1111/acps.13330)
Supplement: Supplementary file 2 — Figure S2 [file ACPS-144-6-s002.docx]

Supplementary Figure 2: Unadjusted Results from Cross-Sectional Studies Reporting Association between Psychosis and Visual Impairment


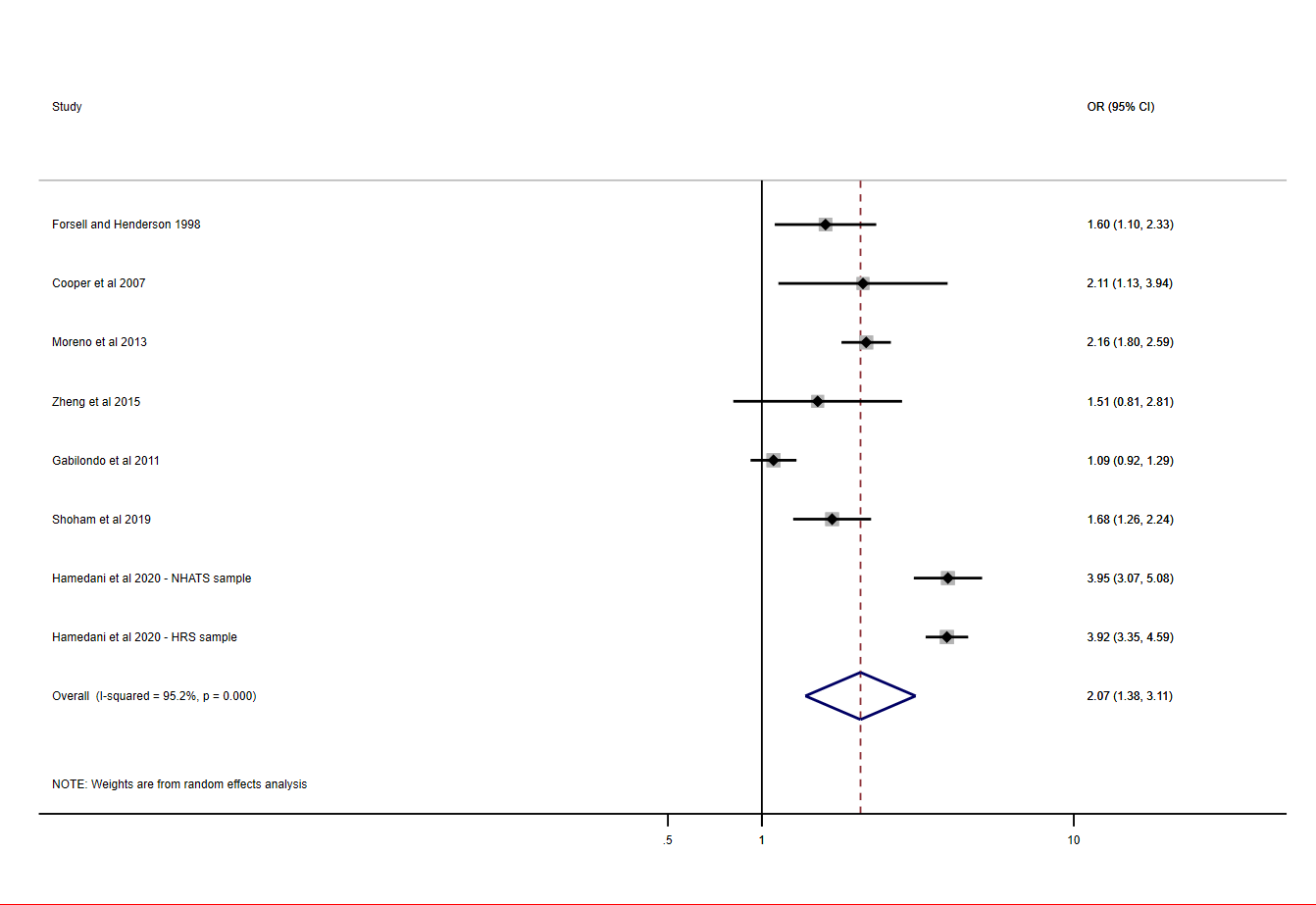


OR = Odds Ratio (unadjusted), 95% CI = 95% Confidence Intervals, NHATS= The National Health and Aging Trends Study, HRS= The Health and Retirement Study
