# Supplementary material for: Associations between psychosis and visual acuity impairment: A systematic review and meta‐analysis
Source: Acta Psychiatr Scand. 2021 Jun 15;144(1):6–27. doi: 10.1111/acps.13330 (PMC8504204; doi:10.1111/acps.13330)
Supplement: Supplementary file 3 — Table S1 [file ACPS-144-6-s004.docx]

Supplementary Table: Study Funding

| **Study** | **Funding Contributors** |
| --- | --- |
| Hayes et al 2018 ^2^ | Medical Research Council  Swedish Research Council |
| Caspi et al 2009 ^48^ | National Institute of Mental Health, Bethesda |
| Stafford et al 2019 ^49^ | Wellcome Trust  Royal Society  Medical Research Council  National Institute for Health Research, University College London Hospital, Biomedical Research Centre. |
| Schubert et al 2005 ^7^ | Stanley Medical Research Institute USA  NIMH USA  Swedish Medical Research Council  Medical Faculty of Lund University, Sweden |
| Schiffman et al 2006 ^6^ | National Institutes of Mental Health  National Institutes of Health |
| Hamedani et al 2020 ^42^ | National Institute of Neurological Disorders and Stroke |
|  | National Institute of Neurological Disorders and Stroke |
| Blazer et al 1996 ^37^ | Not recorded |
| Lee et al 2013 ^51^ | University of Malaya |
| Cumurcu et al 2015 ^52, 57^ | No funding |
| Matsuoka et al 2015 ^44^ | Next Kakenhi |
| Brittain et al 2010 ^53, 74^ | Psychiatry Research Trust  Institute of Psychiatry Psychological Medicine sub-committee |
| Keane et al 2019 ^54-56^ | National Institutes of Health  National Institutes of Mental Health |
| Prager and Jeste 1993 ^19^ | National Institutes of Mental Health  American Council on Aging  Department of Veterans Affairs |
| Schechter et al 2005 ^57^ | National Alliance for Research on Schizophrenia and Depression  Burroughs Wellcome Translational Scientist Award |
| Silverstein et al 2014 ^58^ | National Institutes of Mental Health  National Institutes of Health |
| Almeida et al 1995 ^41^ | Not recorded |
| Saha et al 2011 ^62^ | No funding |
| Shoham et al ^63^ | No funding |
| Livingston et al 2001 ^43^ | Department of Health |
| Zheng et al 2015 ^64^ | National Natural Science Foundation of China  Beijing Nova Program of the Beijing Municipal Science, and Technology Commission. |
| Cooper et al 2007 ^65^ | Greater Glasgow Health Board  West of Scotland R&D Mental Health Programme  Scottish Executive Health Department |
| Kinoshita et al 2009 ^66^ | GlaxoSmithKline International scholarship  Overseas Research Students Awards Scheme  Nitto Foundation |
| Forsell and Henderson 1998 ^39^ | Swedish Society for Medical Research  Swedish Medical Research Council |
| Hamedani et al 2020 ^42^ | National Institute of Neurological Disorders and Stroke |
| Blazer et al 1996 ^37^ | Not recorded |
| Bazant et al 2003 ^35^ | National Institute of Health  University of Nebraska Medical Center, Department of Internal Medicine Summer Undergraduate Research Program |
| Viertio et al 2007 ^3, 75, 76^ | Stanley Medical Research Institute  Academy of Finland  The Yrjö Jahnsson Foundation  The Finnish Eye Foundation |
| Moreno et al 2013 ^1^  Stubbs et al 2016 ^77^  Koyanagi et al 2016 ^78^ | Spanish Ministry of Economy and Competitiveness  Instituto de Salud Carlos III  Centro de Investigación Biomédica en Red de Salud Mental  Madrid Regional Government  European Union Structural Funds  Fundacion Alicia Koplowitz  Fundacion Mutua Madrilena  Network of European Funding for Neuroscience Research)  World Health Organization. |
| Gabilondo et al 2017 ^67^ | No funding |
| Subramaniam et al 2016 ^45^ | Ministry of Health, Singapore  The Singapore Millennium Foundation of the Temasek Trust.  Koyanagi et al:  Instituto de Salud Carlos III–General Branch Evaluation and Promotion of Health Research–and the European Regional Development Fund (ERDF-FEDER) |
| Ballard and Bannister 1995 ^46^ | Not recorded |
| Bayon and Sampedro ^37^ | No funding |
| Ostling and Skoog 2002 ^38^ | Swedish Medical Research Council  Swedish Council for Social Research  Stiftelsen Söderström-Königska Sjukhemmet  Konung Gustaf V: s och Drottning Victorias Stiftelse  Stiftelsen för Gamla Tjänarinnor  Stiftelsen Professor Bror Gadelius’ Minnesfond  The Swedish Society of Medicine  Handlanden Hjalmar Svenssons Forskningsfond  The Göteborg Medical Society  Alma och Anna Yhlen’s Foundation |
| Henderson et al 1998 ^40^ | Australian Rotary Health Research Fund |
