# Supplementary material for: Associations between psychosis and visual acuity impairment: A systematic review and meta‐analysis
Source: Acta Psychiatr Scand. 2021 Jun 15;144(1):6–27. doi: 10.1111/acps.13330 (PMC8504204; doi:10.1111/acps.13330)
Supplement: Supplementary file 4 — Supplementary Material [file ACPS-144-6-s001.docx]

Supplement 1: Search terms

We used the following search terms:

(Visual impairment OR low vision OR visually impaired OR impaired vision OR visual disability OR sight loss OR short-sighted OR myopia OR myope OR myopic OR near-sighted OR refractive error OR eyesight OR visual loss OR vision loss OR partially sighted OR far-sighted OR long-sighted OR nearsightedness OR vision disorder OR farsightedness OR hypermetropia OR hyperopia OR staphyloma OR hypermetrope OR hypermetropic OR hyperope OR ambylopia OR amblyope OR astigmatism OR visual acuity)

AND

(psychosis OR psychotic OR schizophren* OR schizoaffective OR paranoi* OR delusion* OR hallucinat* OR paraphrenia)
